# Effects of ventilation on the indoor spread of COVID-19

**DOI:** 10.1017/jfm.2020.720

**Published:** 2020-09-28

**Authors:** Rajesh K. Bhagat, M. S. Davies Wykes, Stuart B. Dalziel, P. F. Linden

**Affiliations:** 1 Department of Applied Mathematics and Theoretical Physics, University of Cambridge, Centre for Mathematical Sciences, Wilberforce Road, Cambridge CB3 0WA, UK; 2 Department of Engineering, University of Cambridge, Trumpington Street, Cambridge CB2 1PZ, UK

**Keywords:** plumes/thermals, drops, turbulent mixing

## Abstract

Although the relative importance of airborne transmission of the SARS-CoV-2 virus is controversial, increasing evidence suggests that understanding airflows is important for estimation of the risk of contracting COVID-19. The data available so far indicate that indoor transmission of the virus far outstrips outdoor transmission, possibly due to longer exposure times and the decreased turbulence levels (and therefore dispersion) found indoors. In this paper we discuss the role of building ventilation on the possible pathways of airborne particles and examine the fluid mechanics of the processes involved.

## Graphical abstract

**Figure:**
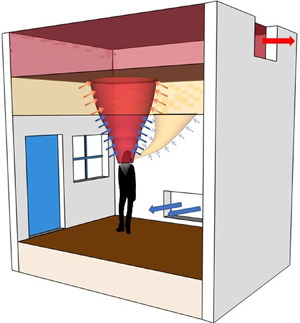

## 1. Introduction

Florence Nightingale, born 200 years ago this year, remarked that ‘the very first requirement in a hospital is that it should do the sick no harm’ (Nightingale 1863). She recommended that wards should have high ceilings, natural light and be well ventilated, principles that guided the design of ‘Nightingale wards’ in UK hospitals for the best part of a century. And, while it is still remains controversial that SARS-CoV-2, the virus causing COVID-19, can be spread by airborne transmission (Fennelly 2020; Lewis 2020; Morawska & Milton 2020; Zhang *et al.* 2020), poorly ventilated places are considered to be high risk and, on a precautionary principle, current advice is for buildings to be as well ventilated as possible (WHO 2020). With the approach of winter and cooler weather in the Northern Hemisphere, where approximately 90 % of the world population reside, there is a clear tension between this requirement and the ability to maintain thermal comfort in buildings without excessive energy consumption.

Since the oil crisis in 1973, and with increasing concerns over the emission of greenhouse gases and climate change, the focus of studies of building ventilation has been on energy efficiency and occupant comfort. This, and generally improved construction standards, has led to tighter buildings and specific ventilation strategies such as mixed-mode ventilation (part natural ventilation, part mechanical ventilation) to reduce the environmental costs of air conditioning in summer. Over the past few years there has been a rising concern about the health impacts of air pollution and, as a consequence, there has been a shift towards considerations of the pollution levels indoors, which taken with temperature and relative humidity constitute indoor air quality (known as IAQ).

This shift in emphasis has come to the fore during the present COVID-19 pandemic, driven by the possibility of infectious aerosols being carried around a building by the ventilation system (Kim *et al.* 2020; Lu *et al.* 2020; Morawska & Milton 2020; Stadnytskyi *et al.* 2020; Zhang *et al.* 2020). A number of outbreaks in confined indoor crowded spaces such as offices, churches, restaurants, ski resorts, shopping centres, worker dormitories, cruise ships and vehicles indicate that virus transmission is particularly efficient in these types of indoor environments (Leclerc *et al.* 2020). Qian *et al.* (2020) studied 318 COVID-19 outbreaks with three or more cases of transmission, and in all except one, the virus transmission occurred in indoor spaces. Park *et al.* (2020) reported an incidence of COVID-19 outbreak in an eleventh-floor office of a call centre in South Korea where 43.5 % of the occupants (94 out of 216 people) were found to be infected; however, the rate of secondary infections to the household members of the symptomatic patients was only 16.2 %. Increased rates of transmission occur not only for buildings, but also on public transportation where people are likely to be in the presence of an infected person in a crowded indoor space for relatively long periods of time and, therefore, exposed to airborne particles (e.g. Hu *et al.* 2020). There is also clear evidence that poor ventilation contributes to the spread of other airborne diseases, such as tuberculosis and SARS (Li *et al.* 2007).

In this article, we examine the role of ventilation on the distribution of airborne contaminants in a space. The primary aim of building ventilation is to provide fresh air for breathing and to remove unwanted heat and contaminants from a space. In winter, there is little unwanted heat and the main requirement is to provide fresh air – the industry recommended rate is 10 litres per second per person (l.s.p.). In summer, this flow is generally not sufficient to remove heat generated within a space by the occupants, equipment and solar gains, and higher ventilation rates or mechanical cooling are often employed, particularly in modern buildings.

The importance of ventilation is expressed in the Wells–Riley equation, which states that the probability 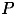 of airborne transmission of an infectious agent indoors is
1.1
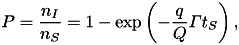

where 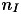 is the expected number of people who become infected by being in the room, 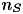 is the number of susceptible people present in the room for a period 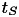, 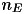 is the number of people emitting infectious ‘quanta’ (describing the mean viral load required for infection) at a rate 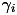 (giving the total emission rate 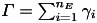), 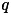 is the time-average volume flux of exhaled air per person and 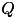 is the volume flux of fresh (uncontaminated) air entering the room (Riley, Murphy & Riley 1978). This assumes that ventilation is uniform across a space. However, as we shall see, local flows can be significant.

**Figure 1.**
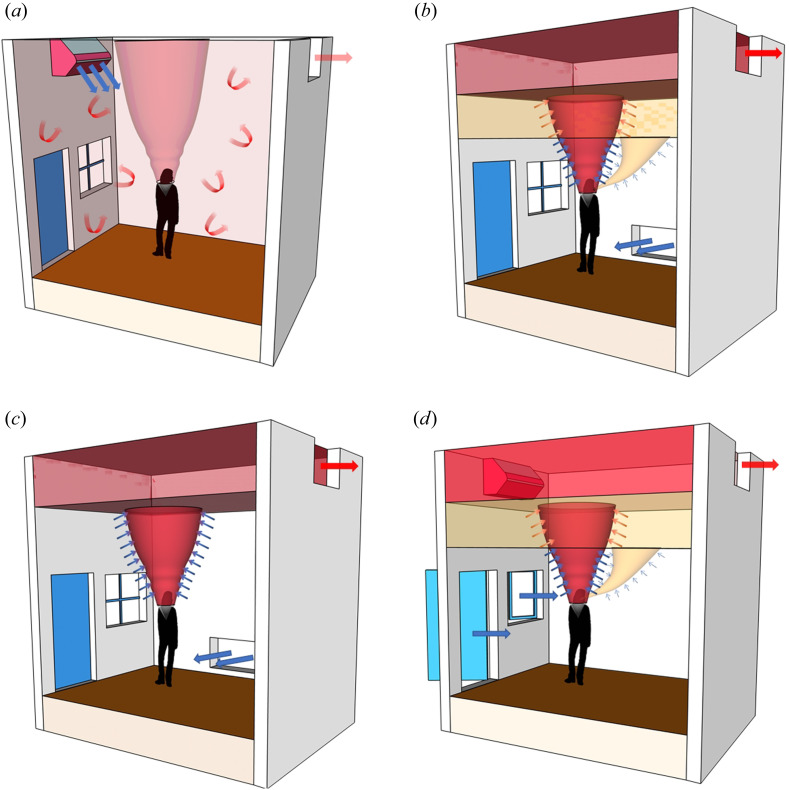
Schematic illustrating ventilation flows with the various flow elements such as the body plume, inlet flows, stratification and arrows indicating entrainment and mixing. (*a*) Mixing ventilation, the hot air rises to the ceiling and, except near the inlets and vents, the indoor conditions remain approximately uniform. (*b*) Displacement ventilation when the occupant does not wear a mask. The secondary breathing plume stratifies below the hot upper layer, and the fluid in the secondary layer gets entrained into the body plume and exhausted out of the indoor space. (*c*) Displacement ventilation when the occupant wears a mask. In this scenario, near its origin, the breathing plume gets caught into the body plume and exhausted out from the upper layer. (*d*) When we turn off the mechanical ventilation input and instead open the doors and windows of space with a top-level opening, ignoring the effect of the wind, it effectively creates a displacement ventilation scenario (here shown when no mask is worn).

Ventilation, whether natural ventilation or mechanical ventilation, has two main modes. The most common, certainly in air conditioned buildings, is mixing ventilation where inlets and outlets are designed to generate flow that keep a space well mixed so that the temperature and any contaminants are uniform throughout the space. The other extreme is displacement ventilation, in which the vents are arranged so that an interior stratification is established with a cool lower zone beneath a warm upper zone. In displacement ventilation, the system is designed to keep occupants in the cool lower zone and so extractors are located in the upper part of the space. A schematic showing these modes is given in figure 1. We discuss the implications for airborne transmission in these ventilation modes and the impacts of other factors important to air movement in buildings. In particular, we show that a significant amount of bio-aerosol expelled during exhalation can remain airborne and be carried around the building by the ventilation flow. Since carbon dioxide is also exhaled and carried by the ventilation flow we propose that concentration levels of 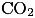 can be used to indicate the potential presence of SARS-CoV-2 in the air, and that high levels should trigger remedial action to reduce the risk of infection.

## 2. Droplets

Transmission of respiratory diseases occurs via expiratory droplets produced by coughing, sneezing, speaking, singing and laughing (Stelzer-Braid *et al.* 2009; Yan *et al.* 2018). Human exhalation contains droplets in the range 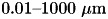 (Bake *et al.* 2019). The medical infectious disease community divides droplets into two classes: droplets larger than 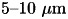 in diameter are classified as respiratory droplets, whereas droplets smaller than 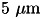 are referred to as aerosols (WHO 2014; Milton 2020). Droplets are considered to fall quickly to the floor close to the source, whereas aerosols are expected to remain airborne for long times. The cutoff between respiratory droplets and aerosols is somewhat arbitrary: in practice, droplets larger than 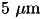 can remain in suspension for long times allowing them to be recirculated within or removed from the room.

Airborne transmission occurs when a person is exposed to an infectious load of pathogen-laden aerosols. Droplets and bio-aerosol produced due to intermittent violent expiratory events such as coughing and sneezing have attracted much attention (Bourouiba, Dehandschoewercker & Bush 2014; Bourouiba 2020), and this subject has recently been reviewed by Mittal, Ni & Seo (2020). However, under normal circumstances, the cumulative amount of bio-aerosol produced by low frequency violent intermittent events of coughing and sneezing is much less than that of breathing and talking. Compared with a person intermittently coughing every minute, in the same period the simple act of breathing or talking produces 10 times the amount of exhaled air (Gupta, Lin & Chen 2010). Furthermore, recent evidence suggests that asymptomatic/presymptomatic airborne transmission, particularly in crowded indoor environments, cannot be ruled out (He *et al.* 2020; Leclerc *et al.* 2020; Park *et al.* 2020; Qian *et al.* 2020).

The infectivity of COVID-19 patients peaks before the onset of symptoms, and preventing presymptomatic and asymptomatic transmission is the key to contain the spread of the virus (Matheson & Lehner 2020). At the early stage of the disease, upper respiratory tract symptoms and the presence of high concentrations of SARS-CoV-2 virus in oral fluids are common (Wölfel *et al.* 2020), supporting recent findings identifying speech droplets to be a potential cause of transmission (Stelzer-Braid *et al.* 2009; Anfinrud *et al.* 2020; Stadnytskyi *et al.* 2020). Conversational speech produces a wide range (submicron up to 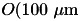) of droplets) while the majority of aerosol particles in exhaled breath are 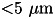 (Fennelly 2020). However, the viral load associated with different aerosol sizes is unknown, making estimates of infectivity, required as input to (1.1), very difficult.

When droplets are exhaled they evaporate at a rate that depends on droplet size and composition, and the relative humidity and temperature of the air. Redrow *et al.* (2011) compared the evaporation time and resulting nuclei sizes of model sputum, saline solution and water droplets. They showed that sputum droplets containing protein, lipid, carbohydrate, salt and water leave larger nuclei than salt solution. They also calculated the time scales of evaporation of water droplets at room temperature, for relative humidities between 0 % to 80 %, to be 0.1–1 s for droplets less than 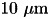 and 7–40 s for 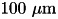 droplets. Therefore, it is expected that droplets larger than 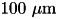 settle on the floor or other nearby surfaces (Liu *et al.* 2017), while droplets smaller than approximately 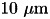 tend to form nuclei and are transported as passive scalars (Xie *et al.* 2007).

The final size of exhalation droplets depends upon many factors including the initial size, non-volatile content, relative humidity, temperature, ventilation flow and the residence time of the droplet. Marr *et al.* (2019) gave the equilibrium size for 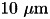 sized model respiratory droplets containing 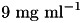 NaCl, 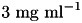 protein and 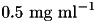 surfactant to be 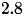 and 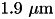 at relative humidities of 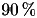 and 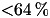, respectively.

## 3. Some numbers

We begin the discussion of ventilation by considering some typical flows in a space. Consider a (generous) one-person office of floor area 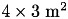 with a floor to ceiling height of 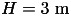. A person gives off approximately 80 W of heat in the form of a convective plume that rises towards the ceiling. The person breathes at a rate of 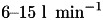 (Gupta *et al.* 2010) and this exhaled breath carries 2.5 %–5 % of the body heat. The recommended ventilation rate is 10 l.s.p., which is equivalent to one air change per hour (ACH) for this room and is much greater than the volume of air breathed, but is needed because the concentration of 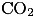 in exhaled breath is around 40 000–53 000 parts per million (p.p.m.). Note for comparison that background external 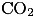 concentrations are currently approximately 415 p.p.m.

In practice, ventilation rates are set between 5–10 ACH, although higher values are used in specialised facilities such as operating theatres. The speed 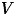 of volume-average flow in the room is 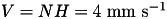 for 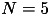 ACH (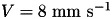 for 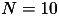 ACH) and this average flow is hardly perceptible to an occupant. To put this in perspective, the Stokes settling velocity of a 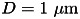 droplet is 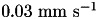, while the asymptotic fall speed of a 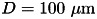 droplet is 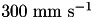. Droplets with 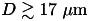 have a settling velocity greater than the volume-average ventilation velocity, even at 10 ACH and, ignoring any evaporation or air movement, the fall time for a 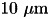 droplet from the release height of 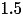 m is 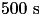 reducing to 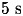 for droplets 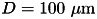. Evaporation, however, means that the 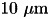 droplet will take longer than this to reach the floor, and indeed may not do so as its settling velocity decreases rapidly as it loses mass.

However, this is by no means the whole (or even the main) story. In practice, air is introduced through a vent or a window that is usually quite small compared with the floor area. For example, the average speed of flow through a vent measuring 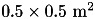 is 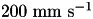 for 5 ACH, much greater than the volume-average velocity. If this is a ceiling vent, the resulting jet (ignoring buoyancy) will still have a velocity of 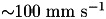 when it reaches the floor. Similarly, the plume above a person giving off 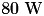 is also approximately 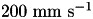, again much greater than the volume-average flow.

Consequently, the air flow patterns within a space are crucial for determining the distribution, transport and fate of any airborne contaminants. Predicting these flow patterns is extremely challenging since they depend critically on both the boundary conditions (e.g. the location of inlet and outlet vents) and on the internal dynamics of the fluid, particularly buoyancy forces associated with temperature differences. This should be contrasted to, say, aerospace where flow round an aerofoil does not depend on the dynamics of the air, and geophysical fluid dynamics where boundary conditions are often unimportant. Further, flows in buildings and other enclosed or semi-enclosed spaces often take place in very complex geometries, making computation of these turbulent flows particularly challenging.

## 4. Ventilation systems

We will now summarise various typical forms of ventilation: mixing ventilation, natural and mechanical dispacement ventilation, and wind-driven ventilation.

### 4.1. Mixing ventilation

In mixing ventilation, the concentration 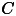 of aerosol suspended in the air is, by design, uniform, and in the absence of any continued input of aerosol satisfies
4.1
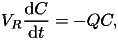

where 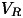 is the room volume and, as in § 1, 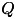 is the volume flux of fresh (uncontaminated) air. In a mechanical system, 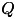 can be assumed constant and the concentration decays exponentially with a time scale 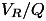. A reduction of the initial concentration by a factor of 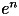 then takes time (in hours) 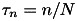, where 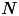 is the number of air changes per hour. For example, a decrease by a factor of 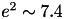 takes 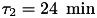 (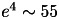 would take 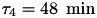) for 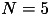 ACH.

If there is a source of infection in the room, we can extend this and relate it back to the Wells–Riley equation (1.1) by adding the total emission rate 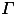 as a source term to the right-hand side of (4.1), giving
4.2
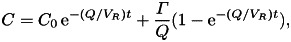

and requiring a time of 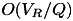 to attain the equilibrium concentration 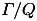 from an initial concentration 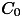 at 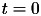.

For a known emission rate 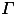, (4.2) gives the concentration of aerosols in a mixing ventilation scenario, which can subsequently be used to calculate occupant exposure. However, knowing the source strength, 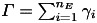, is challenging due to the inherent variability associated with the source physiology, pathogen concentration at the source, physical properties of the exhaled aerosol, and the relative humidity and the temperature of the indoor space. Nevertheless, in practice, 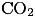 concentration calculated from (4.2) can be a good indicator of the presence of bio-aerosols produced by the occupants (Rudnick & Milton 2003).

### 4.2. Displacement ventilation

In displacement ventilation, on the other hand, the goal is to minimise mixing within the lower occupied zone, allowing the heat and contaminants to rise to the top of the space where they are extracted through upper-level vents. The lower occupied zone is supplied with fresh, uncontaminated air through vents located near the bottom of the space, as illustrated schematically in figure 1. In practice, these ‘low-level’ inlets can be windows or doorways, provided there are high-level outlets available.

#### 4.2.1. Natural displacement ventilation

In stack-driven natural ventilation, warm buoyant air (due to body heat and the heat generated by solar gains, equipment and appliances) rises towards the ceiling and exits through an upper-level opening. This, in turn, draws in cooler (higher density) outdoor air that flows across the floor of the room. The stratification produced by the indoor temperature gradient drives the flow inside the building (figure 1*a*). The average flow is upwards, removing airborne contagion away from the occupants towards the ceiling, where it gets flushed out of the building. The stratification resulting from a single constant heat source consists of two layers, each of uniform temperature, with an interface at a height 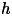 separating the cool unpolluted region below from the warm polluted region above (Linden, Lane-Serff & Smeed 1990).

In practice, heat sources (such as a person or a piece of equipment) produce plumes released from various heights in the space, and the volume of the room below the lowest heat source plays no role as it contains air at the ambient outdoor temperature. Thus the effective height of the room is 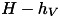, where 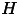 is the floor to ceiling height and 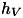 is the ‘virtual origin’ of the lowest plume (i.e. the height at which the plume would start if it was a pure plume of buoyancy from a point source). In practice, it is quite difficult to determine 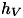 and we return to this issue in § 6.

In the case of 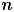 occupants (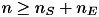), represented by equal strength plumes with the same virtual origin heights, the interface height is independent of the strength of the heat sources and is determined solely by the amount of open area according to
4.3
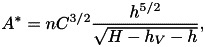

with the empirical constant 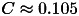 (Morton, Taylor & Turner 1956; Linden 1999). This effective openable area 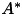 depends on a combination of the total areas 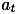 and 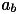 of the top and bottom openings, respectively, given by the relation
4.4
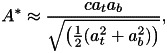

where 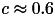 is a discharge coefficient that accounts for flow contraction and the pressure losses at the openings. Note that when the top opening is small (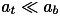), 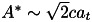, implying that the flow is controlled by the smaller opening. In practice, this allows the interface height to be controlled by a centrally operated upper opening. From (4.3), it is clear that, as noted by Florence Nightingale, buildings with high ceilings and with large openings are optimal for natural displacement ventilation.

#### 4.2.2. Mechanical displacement ventilation

Buildings usually have lower-level openings such as windows and doors, but often lack large upper-level openings. In situations where the required opening area is not available or the space is not tall enough, natural ventilation can be supplemented or replaced by mechanical extraction from the upper part of the space. In this case the height 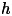 of the lower clean zone is determined by matching the total extraction rate 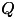 with the flow of warm air from the occupants, etc., into the upper warm zone. For 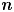 occupants, this is given by the formula
4.5
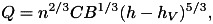

where 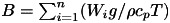 is the buoyancy flux produced by 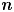 sources with heat fluxes 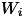, 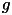 is the acceleration due to gravity and 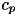 is the specific heat of air. In air a heat flux of 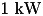 corresponds to 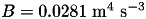. Note that now the height of the space is no longer important, but the depth 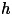 of the clean zone depends on the heat input and is set by the extraction rate. In principle, 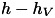 can be set to any height using a suitable mechanical ventilation rate.

In displacement ventilation, the equivalent room volume for removal of a contaminant is simply the volume near the top of the room containing the contaminant. Consequently, the removal time scales are shortened by a factor 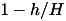 compared with those obtained in mixing ventilation. It is, therefore, advantageous for the interface of the contaminated upper zone to be as close to the ceiling as possible.

### 4.3. Wind-driven ventilation

Wind can also drive natural ventilation in a space, with different models applying for single sided (opening on one side of a space) and cross-ventilation (openings on two sides of a space). Existing models for single-sided ventilation rates driven by wind and buoyancy are based on empirical fits to data from field studies and wind tunnel experiments (Degids & Phaff 1982; Warren & Parkins 1985; Larsen & Heiselberg 2008). These models generally underestimate ventilation rates in full-scale tests (Larsen *et al.* 2018; Gough *et al.* 2020). This is likely to be useful in the context of calculating the time to ventilate a room as this will provide estimates with a safety margin.

The combined effect of wind and buoyancy for cross-ventilation can be modelled using a function of the densimetric Froude number 
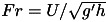
, where 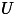 is the wind speed, 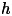 is the opening height, 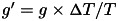, 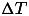 is the indoor–outdoor temperature difference and 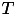 is the room temperature in Kelvin (Davies Wykes, Chahour & Linden 2020). If the incoming flow is sufficiently energetic to mix the space, the concentration of any contaminants will decay exponentially, as in the well mixed case. However, if there is a significant indoor–outdoor temperature difference, the room can stratify, trapping contaminants in the region of the room above the top of the downwind opening, which then remain for long times. A similar trapping effect can occur for buoyancy-driven single-sided exchange ventilation through a window or door (Phillips & Woods 2004).

## 5. Stratification

It is a truism that ‘hot air rises’. In a room, air that is heated by internal heat sources (occupants, equipment, solar radiation, heaters) will rise and tend to accumulate near the ceiling. The well mixed assumption which is implicit for mixing ventilation is only valid if the ventilation is able to mix this warm air throughout the space. Since this process involves moving warm, buoyant air downwards, it requires a source of energy for mixing. Consider the energy required to mix down a layer of air near the ceiling that is 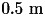 deep and 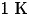 warmer than the air lower in the room. An energy balance (details not given here) assuming a mixing efficiency of 0.2 implies that volume-average velocities of approximately 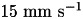 are required. This average flow in the space we are considering requires over 20 ACH! Consequently, for a more reasonable 10 ACH, we expect the space to be stratified even when a mechanical system is introducing cool air at high level.

Typically, heat sources within a space are neither equal nor located at the same height, and the resulting stratification for displacement ventilation is more complicated than the simple two-layer form discussed above. One example is the stratification known as the ‘lock-up effect’, which refers to the possible trapping of exhaled breath below the warm ceiling layer. Since exhaled breath is warm, it rises as a secondary plume and, if it is not immediately entrained into the main body plume, it first settles at an intermediate height and then ultimately is entrained and carried into the upper layer (figure 1*b*). The additional ventilation rate needed to ensure a lower layer of the same height can be calculated by considering the combined effect of two unequal plumes (Cooper & Linden 1996) and is a factor of approximately 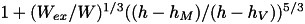, where 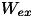 is the heat flux in the exhaled breath, 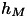 the height of the mouth. Typically, 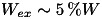 and, for a typical case of 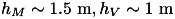 and an upper layer height of 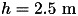, this requires an approximately 23 % increase in the ventilation rate. The size of this increase emphasises that the wearing of face coverings, which block the forward momentum of the exhaled breath and trap it in the body plume, is particularly beneficial as discussed in more detail in § 6.

**Figure 2.**
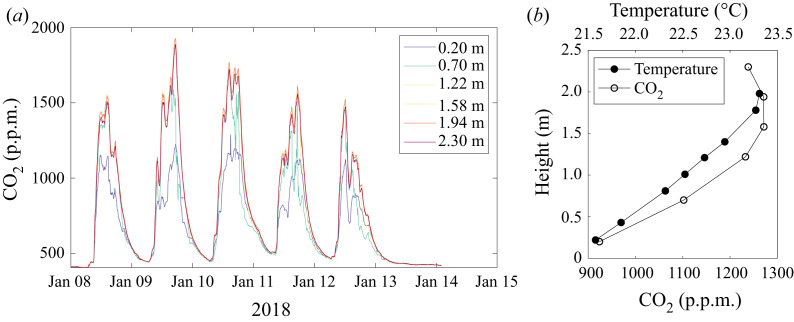
The 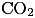 concentration measured at different heights in a naturally ventilated office in London. (*a*) Time variations over five weekdays and (*b*) the mean 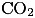 stratification and mean temperature profile during working hours (9:00 to 17:00).

Thermal stratification will not only result in a higher temperature near the ceiling than near the floor, but will also tend to result in a stratification of any contaminants produced by people. Figure 2 shows that 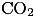 accumulates at the ceiling in a naturally ventilated office even though the density of CO_2_ is approximately 1.5 times that of fresh air. The office also has a stable temperature stratification with the ceiling temperature approximately 2 K higher than the floor (equivalent in density terms to approximately 13 000 p.p.m. 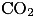), which is more than sufficient to counteract the density of 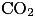 at the measured concentration. Other examples of 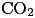 stratification have been reported in mixing-ventilated spaces (Mahyuddin & Awbi 2010; Pei *et al.* 2019).

Stratification can be reduced by the use of ceiling or personal fans, or by the stirring induced by the motion of occupants, which can supply additional kinetic energy to the space. Whether or not stratification is beneficial in a space will depend on the type of ventilation method employed. If the contaminants of concern are carried passively by the flow, then displacement ventilation provides the least contaminated breathing region (Bolster & Linden 2007). However, one concern related to stratification is the possibility that particles originally transported towards the ceiling may settle out of the warm, contaminated air to land on or be inhaled by someone else (Bolster & Linden 2008). Mingotti & Woods (2015) showed there were several regimes for transport of heavy particles when they were introduced into a plume in a displacement-ventilated space. In steady-state, particles with a settling speed smaller than the volume-averaged velocity, 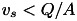, are well mixed throughout the upper layer and any particles settling into the lower layer are re-entrained by the plume. However, for particles with larger settling velocities, a fraction 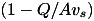 of the particles added to the space will not be transported out of the space, but will instead settle to the floor. This process will be additionally complicated by the evolution of a droplet distribution by evaporation. Upwards transport of aerosols in the body (and other) plumes in the space is a critical and generally unaccounted for feature when modelling the likely exposures of occupants of a space. This topic will be discussed in the next section.

## 6. People

Building occupants are often a source of trouble for designers and building managers. One room temperature does not suit all and, consequently, occupants often complain that it is too hot or too cold, and mess with thermostats and windows in ways that designers had not intended. From the fluid mechanical viewpoint, they can also be a source of considerable complication. As mentioned above, body heat causes a plume to rise above a person and its form and strength depend on body weight and metabolism, posture, the amount and type of clothing, activity level and even hair style. Weak turbulence and other airflow within the room will buffet the plume, causing it to meander as it rises, increasing the entrainment into the plume (Hübner 2004). As mentioned above, displacement-ventilation models are based on the height of the ‘virtual origin’ of the plume – the height at which the actual plume would have started if it were heated by a point source, and this is often difficult to estimate.

### 6.1. Body and breath plumes

**Figure 3.**
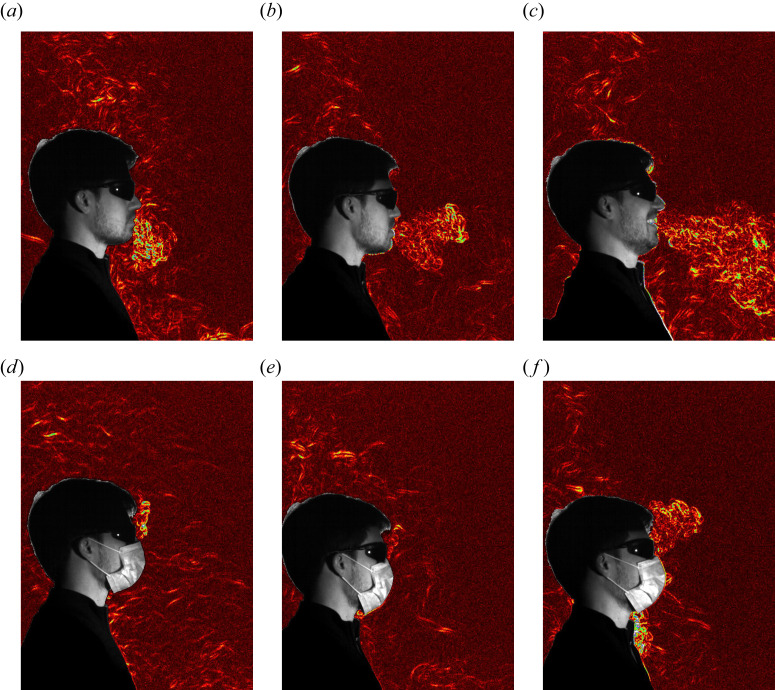
Differential synthetic schlieren images of the thermal plumes produced by a person in a quiescent environment. In panels (*a*–*c*) no mask is worn, while in panels (*d*–*f*) a non-surgical three-ply disposable mask is worn. The subject is (*a*,*d*) sitting quietly breathing through their nose, (*b*,*e*) saying ‘also’ when speaking at a conversational volume and (*c*,*f*) laughing. Video sequences of each of these can be found in the supplementary material.

The body plume and the interaction with exhaled breath can be visualised by observing the temperature-induced refractive index variations in the air using differential synthetic schlieren (Dalziel, Hughes & Sutherland 1998, 2000; Dalziel *et al.* 2007). Figure 3 shows the qualitative version of this technique with images capturing 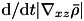, where 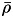 is the line-of-sight mean density and 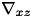 represents the gradient normal to the line of sight. The choice of this diagnostic emphasises the breath of the subject relative to the plume from the body. Here, the test subject is dressed in jeans and a long-sleeved jumper. In figure 3(*a*–*c*) no face covering is worn and the subject is breathing out through their nose (figure 3*a*), speaking at a conversational level (figure 3*b*) and laughing (figure 3*c*). In figure 3(*d*–*f*) the test subject exhibits the same breathing patterns wearing a mass-produced three-ply disposable non-surgical mask (complying with EN14683:2019). In all images the thermal plume driven by the heat flux from the surface of the body is visible, gently wafting upwards. As the test subject is seated, part of this heat flux drives convection from the subject's legs, positioned out of view beneath the image, although here the clothing makes this signal weak. The plume from the body surface is relatively gentle but turbulent and entrains the quiescent ambient air as it rises. Although representing only 2.5 %–5 % of the total heat flux, the thermal signal from the various breathing patterns is clear and, in the absence of a mask, the resulting flow, which will carry the majority of the infectious droplets, follows a different evolution to that of the thermal plume from the body. Video sequences of each of these scenarios may be found online in the supplementary material available at https://doi.org/10.1017/jfm.2020.720.

In the absence of a face covering, an exhaled breath takes the form of a buoyant starting jet. Exhalation from the nose (figure 3*a*) is typically inclined downwards and the air is around 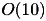 K above room temperature. Mean exhalation velocities are around 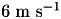 over a period of around 4 s, giving a specific momentum flux 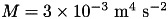 and a buoyancy flux 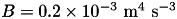. The length before the buoyancy dominates the initial momentum, known as the jet length, is given by 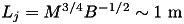. So even though the breath is directed downwards, we expect its momentum is sufficient for it not to be entrained directly into the body plume. (Here, as the subject is sitting, the nasal breath interacts with air rising from the legs, allowing more of it to be entrained into the body plume than would be the case if the subject was standing.) On the other hand, as shown in figure 3(*d*), a face covering significantly reduces the momentum and most of the exhaled breath is then entrained into the body plume and carried upwards.

When speaking, the shape of the oral cavity, air flow and aperture of the lips all change rapidly, leading to source conditions that change for each syllable in a word or sentence. A mouth opened widely imparts little momentum on the (turbulent) air that is emitted, leading to a thermal signal that rises nearly vertically from the point of exit. Some consonants such as ‘b’ or ‘p’ can lead to sudden ejections of air that roll up to form turbulent puffs or vortex rings with significant horizontal momentum, while others such as ‘f’ or ‘v’ direct the flux downwards as a strong jet in an orientation similar to that of the nasal emissions. Figure 3(*b*) illustrates part of a spoken phrase. Here the phrase ‘There were also…’ has just been spoken, with the two distinct structures visible, propagating at different angles, come from the word ‘also’. Specifically, the ‘al’ sound produced a buoyant starting jet directed horizontally that was then reoriented to a more downward trajectory as ‘so’ was sounded. We may reasonably expect the droplet content of the air emitted to also depend on the phrase that is spoken, leading to complex patterns of droplet emission and distribution from normal speech, with some utterances being incorporated into the body plume while others undergo significant dilution before merging with the lock-up layer.

Of the utterances shown here, laughing produces the greatest air and heat fluxes, although without direct measurements we are unable to compare the droplet fluxes with those of speaking. What is clear, however, is that the jet produced from a laugh (figure 3*c*) has significant momentum that carries it down and away from the test subject, diluting it and preventing it from being entrained directly into the body plume. The degree of entrainment in this jet is such that it will be less buoyant than the air exhaled from speaking or normal breathing by the time it reaches its equilibrium height, and so will reside at the bottom of the lock-up layer.

Whereas nasal breathing and speaking produced quite distinct patterns of transport for exhaled breath when the test subject was not wearing a facial covering, there is relatively little to distinguish the distribution of exhaled air in the two cases when a non-surgical three-ply disposable mask is worn (figure 3*d*,*e*), although the duration of the airflow pulses differ. In both cases, the dominant flow of exhaled air is through the gap between the nose and mask, an issue common with many mask designs, with a low-momentum plume forming that hugs the forehead before merging with the body plume. There is little if any leakage evident from other edges of the mask, and any transport of warm, moist air through the mask itself is minimal. In these cases, it is reasonable to expect that the exhaled air will end up in the same layer as the majority of the heat from the surface of the body to form the main ventilated layer at the top of the room.

The case of a laugh, however, is a little different from normal breathing or speaking. The higher volume flux still primarily exits the mask through the gap at the top (this gap is likely to be slightly larger due to the increased pressure associated with the airflow), although some of the flux can also be seen leaking from the bottom of the mask. The leakage from the top now has a more jet-like character to form an upward-directed buoyant jet that separates from the forehead. This separation prevents it from being entrained directly into the body plume. The leakage from the bottom of the mask, however, hugs the body and ends up being incorporated into the body plume. Additionally, some flux through the mask itself is visible in front of the mask, although this is much smaller than the flux at the top of the mask and will have a lower proportion of larger droplets due to the filtering of the mask.

### 6.2. People movement

A person walking through a building has a significant wake. For example, walking at a moderate pace of 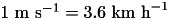 implies Reynolds numbers based on the girth of an average person are 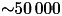 at full scale, implying the wake is turbulent. The wake velocity is approximately 80 % of the person speed, implying flows behind a person of the order of 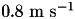 are possibly the largest in a space, capable of resuspending material deposited on surfaces and transporting airborne particles. Experiments on a cylinder passing through an air curtain in a doorway, show that the air curtain is strongly disrupted by the passage and a large volume is transported through the doorway (Jha, Frank & Linden 2020*a*; Jha *et al.* 2020*b*). Enhanced longitudinal dispersion by the repeated movement along a corridor has also recently been reported (Mingotti *et al.* 2020).

**Figure 4.**
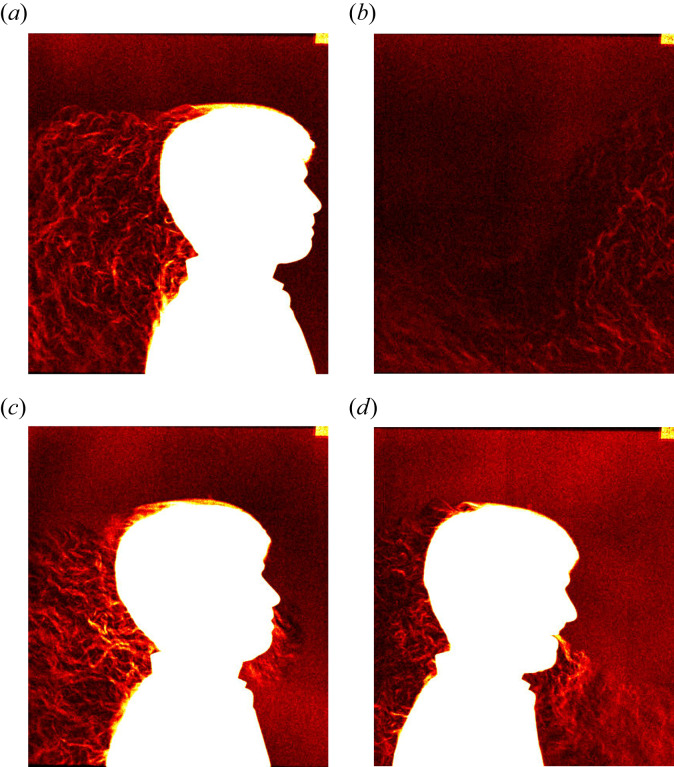
Synthetic schlieren images of the forced convection and thermal wake produced by someone walking slowly through a quiescent room. (*a*) Thermal convection is not visible in front of the person but a thermal signal is clearly visible in the high-Reynolds-number wake of a person walking without breathing. (*b*) The thermal signature of the wake one second after the passage of the person. Significant mixing has occurred without obvious effects of buoyancy. (*c*) Air exhaled by nasal breathing is swept around the head by the motion and entrained into the wake. (*d*) Although laughing still produces a jet that reaches in front of the subject, it is soon overtaken and the associated breath is incorporated into the wake.

The motion of the person also has a profound effect on the structure of the thermal plume generated by the body. Rather than natural convection leading to a coherent thermal plume rising from a localised source, the forced convection imposed by the motion sheds much of the heat flux from the body into the inertially dominated wake. Figure 4(*a*), generated using synthetic schlieren to visualise 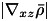, shows clearly this asymmetry with no temperature fluctuations in front of the person, the thermal boundary layer separating at the top of the head (there is a similar separation from the side of the head, but that is not directly visible in the visualisation), and a complex thermal structure in the wake behind them. The mixing that occurs in this wake distributes the (slightly increased) heat flux over a significantly larger volume of fluid, which is less strongly affected by buoyancy and so remains lower in the space for longer periods (figure 4*b* shows the thermal signature one second after the passage of the person remains confined at a level below the top of the head). Although the person in figure 4(*a*) is not exhaling, the motion of the person through the air also has a noticeable effect on how their exhaled air is incorporated into the overall thermal structure of the room. Figure 4(*c*) shows that the jet from nasal breathing is quickly swept back around the person to be incorporated into the wake, whereas figure 4(*d*) demonstrates that while a laugh has the momentum to extend some distance in front of the person, it may still end up being entrained into the wake if the person does not change direction.

In addition to the flow induced by the wake of a person, transient effects occur when a person enters or leaves a space. How long does it take after a person enters an initially unoccupied space for a steady state to be established? The relevant time scales are set by the ventilation time scale 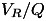, and the ‘filling-box’ time 
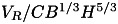
 (Baines & Turner 1969), which is the volume of the space divided by the volume flux in the body plume at the top of the space. The ratio of these time scales 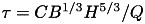 determines whether the steady state is determined by the ventilation rate (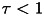) or by the buoyancy-driven flow (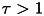). For a single person 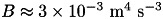 and in a 3 m high room, the transition 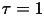 corresponds to 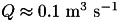 (or 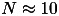 ACH in our office) so in practice the time scale is usually set by the convective heat flow from the occupant. In a lecture theatre holding 100 people the transition ventilation rate is 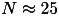 ACH, so again the relevant time scale is the ‘filling box’ time. For a lecture theatre measuring 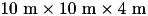, this time is approximately 600 s.

## 7. Closing thoughts

We have shown room flows are ‘turbulent’ in the sense that spatiotemporal variations of the flow are larger than the mean flow. They take place in complex geometries where the placement and sizes of inlets and outlets determine overall flow patterns, superimposed on which are significant perturbations associated with often transient events such as the movement of occupants, the opening and closing of doors, and (for naturally ventilated buildings) variations in the external conditions. The dispersal of a second phase in such an environment is complicated, as droplets are released over a continuum of sizes and they evaporate and reduce in size with time. However, our analysis suggests that airborne transmission of the virus can occur in particles with fall speeds that are lower than typical velocities found in the room and so are advected through the space effectively like a passive tracer.

In that case it seems reasonable to consider 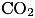 as a marker for air that has been exhaled. Indeed, it has been shown that 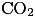 concentration can be linked to the probability of infection predicted by the Wells–Riley equation (1.1) (Rudnick & Milton 2003). Even though 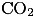 is denser than air, our observations show that it is carried with the flow as would virus particles. A simple balance of a person breathing out at a concentration of 45 000 p.p.m. at a rate of 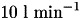 and supplied with the recommended 10 l.s.p., implies that a steady concentration above the background would be 750 p.p.m. Carbon dioxide concentrations above this value, especially at the breathing level, may indicate that the ventilation is inadequate and that remedial action should be taken. The risk of infection is thought to increase with exposure time. It is also the case that 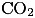 levels increase over time once people begin to occupy a space. Consequently, it may be appropriate to add some exposure time as well as simply the 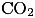 concentration level to a warning system.

Despite the various mechanisms generating disturbances indoors, it is clear that in many cases stratification ‘wins’. A small temperature difference across a doorway or window will organise the flow so that the cool air flows through the lower part and warm air through the upper part of the opening (Linden & Simpson 1985). It is notoriously difficult to mix a stratified space with mixing efficiencies (the ratio of the kinetic energy needed to change the potential energy required) typically well below 20 % (Linden 1979). The presence of stratification emphasises the need to measure 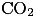 at a height where individuals are breathing, and away from sources of fresh air such as an open window, where concentrations are typically much lower than the room average, if one is to obtain an estimate for the load of potentially infectious particles.

Consequently, if designed properly, displacement ventilation, which encourages vertical stratification and is designed to remove the polluted warm air near the ceiling, seems to be the most effective at reducing the exposure risk. Mixing ventilation distributes the air throughout the space and does not provide any potentially clean zones. It also has to work against the tendency of the room to stratify, while displacement ventilation takes advantage of it, and can simply and cheaply be implemented by installing extraction vents or fans at the top of the space. However, there remain some interesting questions on the behaviour of lock-up layers, particularly regarding the behaviour of particulates in the flow that need to be understood to optimally configure the system.

Our observations show that face coverings are effective at reducing the direct ejection of breath and bio-aerosols away from the person and, when wearing a mask, the majority of the breath is entrained into the body plume. However, many questions remain about aspects of this and other issues discussed in this paper. For example, when a person is moving their wake also entrains part of the breath and the body plume, but how this partition depends on walking speed is unknown, as is the stirring effect of a walking person on the stratification in a room or corridor. The interaction of wakes and body plumes of people passing each other is unexplored: is it different in a narrow corridor compared with an open plan office? The role of other buoyancy-driven flows such as circulations set up by open refrigerator shelves in a supermarket on aerosol dispersion is unknown. Further, the conditions when stratification is established (or destroyed) are only known in a few cases.

We have described here just some of the many flows that are relevant to dispersion of aerosols indoors. We hope the examples we have described demonstrate some of the fascination of fluid mechanics, as well as its applicability to this pressing societal problem.

## Supplementary material

For supplementary material accompanying this paper visit https://doi.org/10.1017/jfm.2020.720. click here to view supplementary material
